# Unintended Sunburn: A Potential Target for Sun Protection Messages

**DOI:** 10.1155/2017/6902942

**Published:** 2017-04-03

**Authors:** Geraldine F. H. McLeod, Anthony I. Reeder, Andrew R. Gray, Rob McGee

**Affiliations:** ^1^Christchurch Health and Development Study, Department of Psychological Medicine, University of Otago, Christchurch, New Zealand; ^2^Cancer Society Social & Behavioural Research Unit, Department of Preventive & Social Medicine, University of Otago School of Medicine, Dunedin, New Zealand; ^3^Department of Preventive & Social Medicine, University of Otago School of Medicine, Dunedin, New Zealand

## Abstract

New Zealand (NZ) has the highest melanoma incidence rate in the world. Primary prevention efforts focus on reducing sunburn incidence and increasing sun protective practices in the population. However, sunburn from excessive ultraviolet radiation (UVR) remains common. To reduce sunburn incidence, it is important to examine those individuals who experience unintended sunburn. This study aims to use data from the NZ Triennial Sun Protection Survey to describe respondents who were not intending to tan but were sunburnt after outdoor UVR exposure. Information on sociodemographics, concurrent weather conditions, sun protection attitudes and knowledge, and outdoor behaviour was also collected. The results showed 13.5% of respondents' experienced unintended sunburn during the survey weekend but had not attempted to obtain a tan that summer. Respondents who reported unintended sunburn were more likely than others to have been near water and in unshaded areas, used sunscreen, had higher SunSmart knowledge scores, had lower positive attitudes towards tanning, and were outdoors for a longer duration with less body coverage. As sunburn was unintended these respondents' outdoor sun protective behaviours may be amenable to change. Future public health initiatives should focus on increasing sun protection (clothing and shade) and reducing potential barriers to sun protection.

## 1. Introduction

New Zealand (NZ) has the highest overall incidence rate of cutaneous malignant melanoma (melanoma), the deadliest form of skin cancer [1]. Sunburn and excessive exposure to UVR have been implicated in the pathogenesis of this skin cancer [2, 3]. To combat this problem, Australia developed the renowned SunSmart public health programme which aimed to reduce excessive sun exposure. This programme is reported to have been successful in increasing sun protection behaviours in their population [4–7]. This campaign was subsequently replicated in various countries including New Zealand, the UK, and the US [8–13].

Evaluation of these public health campaigns has resulted in an extensive literature examining population demographic characteristics and prediction models of UVR overexposure, to further aid the reduction of sunburn [9, 14, 15]. However, weekend sunburn incidence among these populations remains common [6, 7, 15–19]. For example, data from Australia showed that in 1994/95 sunburn incidence was 23.1% among the youngest respondents (14–24 years), 13.7% among respondents with 25–44 years, and 10.1% among the oldest respondents (45–69 years), while in the year 2005/06 sunburn incidence ranges were 17.8%, 11.9%, and 7.3%, respectively [7].

Comparable data from NZ has shown that estimates of sunburn from the same time period ranged from 17.0% (1994) to 20.8% (2005/06) for respondents aged 15–69 years [20]. More recently, Kruse [21] reported that weekend sunburn incidence among the New Zealand population was 19.0% suggesting little decline in rates of sunburn over a 20-year period. Although Trowland et al. [22] indicated that a decline may have occurred in the 2016 Sun Exposure Survey, this finding needs to be confirmed by being sustained in subsequent surveys.

New Zealand public health efforts aim to reduce sunburn by encouraging citizens to engage in SunSmart outdoor behaviour including limiting outdoor sun exposure during peak UVR periods, wearing protective clothing and sun protective broad brimmed, bucket, or Legionnaire's hats, using 30SPF+ sunscreen, and wearing sunglasses that meet Australian and New Zealand standards [23–27]. In addition, public health efforts aim to decrease the desirability of tanning or having a tanned appearance [8, 27, 28].

It is possible that, despite these messages, those experiencing sunburn might consist of two distinct groups: those intentionally seeking to tan who were sunburnt from deliberate sun exposure; and those who are not actively attempting to tan but inadvertently became burned through prolonged or unprotected sun exposure.

The reasons for focussing on the unintended sunburn group are threefold. First, the unintended sunburn group may be substantial in terms of the number of individuals experiencing sunburn. Second, the unintended sunburn group may have less positive attitudes towards tanning and may be more amenable to behavioural change to reduce the risk of future unintended sunburns [29, 30]. Third, the unintended sunburn group may have a pattern of high intermittent UV exposure as their sunburn may be a result of a lack of preparation for being outdoors [15, 31]; intense intermittent sun exposure is implicated in the development of melanoma [32–34].

Little is known about unintended sunburn as few studies have examined this issue. One study by Køster [35] reported unintended sunburn as an aside to their main findings. In that study, data was collected on the prevalence of sunburn and sun-related behaviour in a Danish sample of 3,499 respondents. The results showed that 33% of unintentional tanners had been sunburnt in the previous 12 months. While the study authors concluded that “future campaigns to reduce the prevalence of sunburn in the Danish population must especially target…intentional tanning” (p. 548) [35], the study also provides evidence that it may be important to focus on those individuals who, while not actively seeking a tan, became sunburnt unintentionally.

Therefore, while efforts still need to be made to reduce the desirability of tanning, it is now also important to focus on those individuals who, while not seeking a tan, became sunburnt unintentionally. Against this background, this study aims to use the Triennial Sun Protection Survey, a NZ dataset to

1. report the incidence of unintended sunburn in a summer weekend,
2. describe the characteristics of respondents reporting unintended sunburn.

## 2. Methods

### 2.1. Study Design and Participants

Every three years during selected southern hemisphere summer weekends, 1999/00-2006, computer assisted telephone interview (CATI) surveys were conducted among NZ residents, 15–69 years (total population in this age group ranged from 2.6 to 2.9 million during this period) [36]. This Triennial Sun Protection Survey (hereafter, the Survey) was commissioned by the Health Sponsorship Council (now the NZ Health Promotion Agency) and the Cancer Society of New Zealand Inc. Surveys were conducted in randomly selected households of five metropolitan cities (Auckland, Hamilton, Wellington, Christchurch, and Dunedin) immediately following weekends identified as likely to be suitable for erythema in unprotected skin, 11 am–4 pm [37]. A quota sampling system ensured the sample had equal numbers of male and female respondents, with equal representation from each metropolitan area. Respondents who had spent longer than 15 minutes outdoors on either the previous Saturday or Sunday, between 11 am and 4 pm, (daylight saving time NZ Standard Time + 1 hour), were interviewed. Ethical approval for analysis and reporting of the anonymous data collected was obtained from the University of Otago Human Ethics Committee. Respondent participation was taken as informed consent.

### 2.2. Measures

*Unintended Sunburn*. To classify respondents as experiencing unintended sunburn, they needed to report experiencing sunburn (defined as “any amount of reddening of the skin after being in the sun”) during the target interview day and also report they had not attempted to tan during the current summer. Those who were not categorised with unintended sunburn were classified into a group of all the remaining Survey respondents. 

*Demographic Information*. Demographic information included age, highest educational qualification, occupation, self-defined ethnicity (Maori, Pacific, Asian, NZ European/European, or “other” prioritized at the highest level of the NZ Ministry of Health ethnicity data protocols [38]), and self-reported skin type (four Fitzpatrick scale [39] categories modified for use in NZ [28]). Survey year/weekend, city of residence, and sex were identified by the interviewer.

*Weather Information*. The National Climate Database (CliFlo) [40] and UV 2.1 Atlas [41] were accessed for air temperature, wind speed, cloud cover, and ultraviolet radiation (UVR) at each respondent's location for the duration of their outdoor activity on the chosen recall day and merged with the survey data.

*Sun Protection Attitudes and Knowledge*. Respondents rated their sun-tanning perceptions on a Likert-type item (1 = strongly disagree to 5 = strongly agree) to indicate their (dis)agreement with six statements; for example, “my friends think a suntan is good”; “I feel more healthy with a suntan.” These were used to create a summative, unweighted positive perception of tanning (ProTan) scale [28] that has been found to have satisfactory psychometric properties [42]. The Knowledge Index questions included an assessment that the respondent (1) knew the SunSmart program existed, (2) could accurately recall up to five behaviours capable of reducing the risk of sunburn and/or skin cancer, and (3) understood that possession of a tan does not protect from skin cancer (see Online Supplementary Material 1 available online at https://doi.org/10.1155/2017/6902942).

*Outdoor Behaviour*. Respondents' information on main outdoor activity was collapsed into six categories: “active,” “passive,” “water-based,” “unspecified” recreation, and “unpaid” and “paid” employment [20]. Respondents also reported the shade status of their activity (full shade, some shade/sun, full sun), their sunscreen use (yes/no), duration outdoors (minutes), and body coverage by clothing (as a percentage).

### 2.3. Statistical Methods

To identify any differences between those who were classified with unintended sunburn and those who were not, Chi-squared tests of independence were used for categorical variables and independent sample *t*-tests were used for continuous variables (subject to model assumptions being met). Where expected cell frequencies were below 5 for more than 20% of the cells, Fisher's exact test was used in place of the Chi-squared tests. Row percentages were calculated, representing the percentage of those unintentionally sunburned within each category of the measures. For *t*-tests, approximate normality and homoscedasticity for the two groups was determined before performing the tests. All analyses were performed using SAS 9.4 [43]. The level of statistical significance for all analyses was set at *p* < 0.05 with two-sided tests used for comparison of means.

## 3. Results

This study used data from 2480 respondents who were outdoors for 15 minutes or longer on the target day and who had full information available. Overall, 335 of those respondents reported that they had experienced sunburn during the survey weekend but had not attempted to obtain a tan that summer, an incidence of 13.5% (335/2480).

Table 1 shows frequencies of respondents reporting being sunburned unintentionally and respondents who did not report this, broken down by nonmodifiable demographic characteristics and weather conditions experienced, pooled over survey years 1999/00 to 2005/06. The statistically significant differences (all *p* ≤ 0.023) were as follows: those sunburned unintentionally more often reported that they had skin types I and II rather than III and IV and were outdoors during times when there was less wind and higher UVR levels and that it occurred during December. No evidence was noted for differences by sex, age group, city, ethnicity, education, occupation, temperature, or cloud cover and no evidence was found for changes between survey years.

Table 2 shows frequencies similarly broken down by potentially modifiable respondent characteristics. For all measures, statistically significant differences were found between the unintended sunburn group and the others (all *p* ≤ 0.023). Overall, the highest rates of unintended sunburn were among those who were near water and in unshaded areas, used sunscreen, had higher SunSmart knowledge scores, had lower positive attitudes towards tanning, and were outdoors for a longer duration with less body coverage.

## 4. Discussion

The aim of this study was to report the incidence of unintended sunburn among a New Zealand urban population. In this study, unintended sunburn was defined as sunburn during the survey weekend in which the respondent reported that they had not attempted to obtain a tan during the summer. In addition, this study aimed to describe the respondents' demographic characteristics, concurrent weather conditions, and potentially modifiable knowledge, attitude, and behaviour characteristics to determine whether any of these varied between those reporting and not reporting unintended sunburn.

Overall, 13.5% (*n* = 335) of respondents described experiencing unintended sunburn. By contrast, 7.3% (*n* = 182) of respondents reported sunburn which was a result of deliberate tanning (results not shown). Respondents reporting unintended sunburn had lighter skin types and experienced sunburn under concurrent weather conditions of lighter wind speeds and higher UVR. The majority of unintentional sunburns occurred in early summer (southern hemisphere December). A number of potentially modifiable factors were also associated with the experience of unintended sunburn. In particular, unintended sunburn was reported more frequently for those in unshaded areas, who had higher SunSmart knowledge and were outdoors for a longer duration. Those who were unintentionally sunburned also had lower positive attitudes towards tanning and had lower body coverage. Finally, there was some evidence that those who were unintentionally sunburned were near water and had worn sunscreen.

As the unintentionally sunburned group already have lower positive attitudes towards tanning and higher SunSmart knowledge scores, education programmes emphasising changing attitudes and knowledge as a way of inducing SunSmart behaviours may not be especially effective in this group. It is recommended that, for this group, future public health initiatives in this area focus on increasing sun protection, particularly protection through the use of clothing and shade [15, 18, 31]. The findings of this study do not support the use of sunscreen as a form of sun protection against UVR. This recommendation may appear paradoxical; however, previous studies have shown that individuals using sunscreen are more likely to become sunburned [31, 44, 45]. This study also showed no evidence that increasing SunSmart knowledge may help to reduce sunburn incidence. Higher mean SunSmart knowledge was associated with unintended sunburn, a finding also seen in previous research [14, 46]. Overall, these study findings support the idea that unintended sunburning is more common than burning as a consequence of intentional tanning. This suggests that support actions are needed to address barriers to sun protective practices in recreational areas (e.g., increase the supply of attractive shade structures in recreational areas) [9, 15, 18, 46]. Our findings may also partially explain poor results from SunSmart campaigns that rely extensively on attitude and knowledge change [20, 21].

It is difficult to compare the results of this study with previous research, as few other studies have reported rates of unintended sunburn. Køster [35] reported an unintended sunburn frequency of 33%, whereas in the current study it was 13%. There may be some explanations for this apparent large difference. In the Køster study, respondents were asked to report sunburn experienced in the previous 12 months, whereas the recall period for the current study was the previous weekend. Recall from the previous weekend is subject to much less potential recall bias as interviews were conducted within three days of outdoor activity and sunburn occurrence. In addition, the questions on intentional tanning (and unintentional tanning) were more detailed in the Køster study with more exploration of time spent sunbathing and tanning intention. In contrast, the current study assessed unintended sunburn more indirectly as it only had one question on whether the respondent had attempted to tan that summer. Therefore, it is difficult to compare these study results and more research in this area is needed.

The strengths of this study include the large sample-size providing power to detect associations and the minimisation of recall bias due to interviews taking place within three days of outdoor exposure. However, some limitations should be taken into consideration. The data refers to the period 1999–2006, and it is possible that associations have since changed and data may not reflect the current situation. The extent to which the findings may be generalizable to the current total population or other populations is unclear. However, we are unaware of any plausible reason for substantial shifts in associations and no more recent survey data are available to address this particular question. This is because, in 2009, the Triennial Sun Protection Survey was reviewed by the associated agencies and replaced triennially from 2010 onwards by the Sun Exposure Survey [47]. Another limitation of this study is that no direct measure of unintended sunburn was made. Unintended sunburn was recorded when the respondent had reported both sunburn experience on the recall day and no attempt to tan during the current summer. It is possible that a portion of these participants may have had their sunburn misclassified as “unintended” and future research should address the issue of unintended sunburn by developing an item or items that measure unintended sunburn directly.

In conclusion, unintended sunburn may result after encountering barriers to sun protection [15, 18, 46]. Potential barriers to sun protection include lack of available sun protection during outdoor activity or lack of appropriate protective behaviours [9, 15, 18, 46]. Therefore, it is important that the problem of unintended sunburn is investigated so that more effective mechanisms can be found to help protect those who are also trying to protect themselves. With the high levels of sunburn reported internationally and particularly the existence of a large group of people unintentionally becoming sunburned, this should now be a priority area for future investigation.

## Supplementary Material

This Online Supplement describes the development of, and the items used in the SunSmart knowledge Index.

## Figures and Tables

**Table 1:** Respondent demographic, survey year, and weather characteristics for those sunburned unintentionally and those not sunburned (aside from survey year these are pooled over survey years 1999/00 to 2005/06).

Measure	Unintentional sunburn			*p*
	Yes (*n* = 335)	No (*n* = 2145)	% within category yes^g^	
	%^b^ (*n*)	%^b^ (*n*)		
*Sex*				0.425
Male	54.9 (184)	52.6 (1128)	14.0	
Female	45.1 (151)	47.4 (1017)	12.9	
*Age group*				0.616
15–19	10.2 (34)	10.3 (221)	13.3	
20–29	15.2 (51)	15.9 (340)	13.0	
30–39	23.3 (78)	19.8 (424)	15.5	
40–49	22.1 (74)	21.8 (467)	13.7	
50–59	20.3 (68)	20.8 (447)	13.2	
60–69	9.0 (30)	11.5 (246)	10.9	
*Skin type* ^a^				<0.001
I	28.5 (95)	23.9 (509)	15.7	
II	62.2 (207)	57.1 (1214)	14.6	
III	8.7 (29)	17.8 (379)	7.1	
IV	0.6 (2)	1.2 (26)	7.1	
*Missing n*	*2*	*17*		
*City*				0.421
Auckland	20.9 (70)	21.3 (457)	13.3	
Hamilton	21.5 (72)	21.2 (454)	13.7	
Wellington	16.4 (55)	19.7 (423)	11.5	
Christchurch	19.4 (65)	19.8 (424)	13.3	
Dunedin	21.8 (73)	18.0 (387)	15.9	
*Priority ethnicity*				0.308
European	91.0 (303)	86.9 (1853)	14.1	
Maori	4.2 (14)	7.0 (149)	9.0	
Pacific	1.5 (5)	2.0 (43)	10.4	
Asian	2.7 (9)	3.4 (72)	11.1	
All others	0.6 (2)	0.8 (16)	11.1	
*Missing n*	*2*	*12*		
*Highest educational qualification*				0.563
School cert. or less	29.9 (100)	26.3 (563)	15.1	
Year 12-13	20.3 (68)	20.9 (449)	13.2	
Certificate	24.8 (83)	25.5 (546)	13.2	
Other^c^	3.9 (13)	3.2 (69)	15.9	
Degree	21.2 (71)	24.2 (518)	12.1	
*Occupation*				0.173
Sales or white collar	29.3 (98)	28.3 (606)	13.9	
Semiskilled	10.5 (35)	10.4 (223)	13.6	
Skilled	18.5 (62)	14.2 (305)	16.9	
Other^d^	15.8 (53)	15.9 (340)	13.5	
Professional	26.0 (87)	31.3 (671)	11.5	
*Survey month*				<0.001
December	28.1 (94)	22.8 (489)	16.1	
January	27.5 (92)	25.0 (536)	14.6	
February	39.7 (133)	40.6 (870)	13.3	
March	4.8 (16)	11.7 (250)	6.0	
*Survey year*				0.222
1999/2000	33.7 (113)	31.9 (685)	14.2	
2002/2003	28.4 (95)	33.1 (710)	11.8	
2005/2006	37.9 (127)	35.0 (750)	14.5	
*Weather information*				
Mean temperature (°C)	22.3 (2.9)^e^	21.7 (3.1)^e^		0.160^f^
*Missing n*	*7*	*34*		
Mean wind speed (Km/h)	19.4 (8.9)^e^	19.8 (9.8)^e^		0.023^f^
*Missing n*	*7*	*5*		
Mean cloud cover (Octa)	3.6 (1.5)^e^	3.7 (1.5)^e^		0.554^f^
*Missing n*	*99*	*480*		
UVR (joules)	738.2 (161.9)^e^	715.1 (182.3)^e^		0.006^f^
*Missing n*	*1*	*17*		

*Note*. ^a^Fitzpatrick sun-sensitivity scale, modified. ^b^Percentages (may not be total 100% due to rounding). ^c^Other includes highest educational qualification unspecified and not knowing. ^d^Other includes occupations listed as student, unemployed, no main earner, other, and missing. % (*n*) reported except for ^e^mean (SD). Chi-square test for independence reported except for ^f^*t*-tests. ^g^Row percentages were calculated, representing the percentage of those unintentionally sunburned within each category of the measures.

**Table 2:** Potentially modifiable respondent behaviours, attitudes, and knowledge for those sunburned inadvertently and those not sunburned (pooled over survey years 1999/00 to 2005/06).

Potentially modifiable variables	Unintentional sunburn			*p*
	Yes %^a^ (*n*) (*n* = 335)	No %^a^ (*n*) (*n* = 2145)	% within category yes^e^	
*Main activity*				<0.001^b^
Passive recreation	24.2 (79)	19.4 (412)	16.1	
Water-based recreation	19.9 (65)	12.3 (262)	19.9	
Paid work	5.2 (17)	4.0 (84)	16.8	
Unpaid work	7.0 (23)	10.5 (222)	9.4	
Unspecified	1.0 (3)	1.0 (21)	12.5	
Active recreation	42.8 (140)	52.9 (1122)	11.1	
*Missing n*	*8*	*30*		
*Shade status*				<0.001^b^
Part shade	20.9 (70)	21.1 (452)	13.4	
Unshaded	67.8 (227)	59.2 (1265)	15.2	
Shade	11.3 (38)	19.7 (422)	8.3	
*Missing n*	*0*	*5*		
*Sunscreen use*				0.023^b^
Yes	47.6 (156)	40.9 (856)	15.4	
No	52.4 (172)	59.1 (1238)	12.2	
*Missing n*	*7*	*58*		
*Attitudes towards tanning*	13.9 (5.3)^c^	15.7 (5.9)^c^		<0.001^d^
*Missing n*	*1*	*2*		
*SunSmart knowledge*	2.4 (1.5)^c^	2.2 (1.5)^c^		0.017^d^
*Duration outdoors *	163.1 (94.4)^c^	134.3 (87.3)^c^		<0.001^d^
*Body coverage*	59.9 (21.9)^c^	62.9 (21.6)^c^		0.019^d^

*Note*. ^a^Percentages (may not be total 100% due to rounding) except for ^c^mean (SD). ^b^Chi-square test for independence. ^d^*t*-test for independent samples. ^e^Row percentages were calculated, representing the percentage of those unintentionally sunburned within each category of the measures.
