# Supplementary material for: Unintended Sunburn: A Potential Target for Sun Protection Messages
Source: J Skin Cancer. 2017 Apr 3;2017:6902942. doi: 10.1155/2017/6902942 (PMC5394386; doi:10.1155/2017/6902942)
Supplement: Supplementary file 1 — This Online Supplement describes the development of, and the items used in the SunSmart knowledge Index. [file 6902942.f1.docx]

**Online supplement 1 for “Unintended sunburn – A potential target for sun protection messages”**

Supplement for Journal of Skin Cancer

This Online Supplement describes the development of, and the items used in the SunSmart knowledge Index.

Geraldine F.H. McLeod^1^

Anthony I. Reeder^2^

Andrew R. Gray^3^

Rob McGee^3^

^1^ Christchurch Health and Development Study, Department of Psychological Medicine, University of Otago, Christchurch, New Zealand

^2^ Cancer Society Social & Behavioural Research Unit, Department of Preventive & Social Medicine,

University of Otago School of Medicine, New Zealand

^3^ Department of Preventive & Social Medicine, University of Otago School of Medicine, New Zealand

**Online supplement 1:**

**Description of the SunSmart knowledge index**

The SunSmart knowledge index questions included items asking if the respondent: (a.) knew the SunSmart program existed, (b.) was able to recall up to five accurate SunSmart behaviours to reduce the risk of sunburn and/or skin cancer, and (c.) understood that possession of a tan does not protect the respondent from skin cancer. The questions were used as an unweighted summative scale and are as follows:

- ‘Have you seen or heard any recent advertising or information about the danger of too much exposure to the sun?’ (Yes/No).
- ‘What was the message you got from [the previous question]? (recorded verbatim with responses probed to “no”). Responses were coded into the various SunSmart messages to which the New Zealand public had been exposed since the early 1980’s. Possible answers included: using clothing, a hat and/or sunscreen, seeking shade, expressing an understanding of the ultraviolet index (UVI) (formally Burn Time), and that secondary prevention measures of skin cancer are important (i.e., regular skin checks). The respondents were allocated one-point for each correctly recalled SunSmart behaviour and/or risk factor for skin cancer, to a maximum of five-points.
- Response options to the statement ‘A suntan protects you against melanoma and other skin cancers’ were presented on a five-point Likert-type item and respondents were asked to report their level of agreement or disagreement (1=strongly disagree; 2=disagree; 3=neither agree nor disagree; 4=agree; 5=strongly agree) with the statement. The responses were dichotomised into two categories of either agree (including strongly agree, agree, neither agree nor disagree, and disagree) or disagree (including strongly disagree) due to skew in the distributions. Furthermore, this dichotomisation was used to ensure that knowledge that a tan does not protect against skin cancer only included those respondents who recorded the only absolutely correct option – strong disagreement that a tan was protective.

The SunSmart knowledge index ranged from zero to seven-points, where zero represented that the respondent had no SunSmart knowledge, and seven represented respondents with the highest possible SunSmart knowledge scores.
